# Treatment Plan Comparison Between Self-Shielding Gyroscopic Radiosurgery and Robotic Radiosurgery

**DOI:** 10.7759/cureus.82990

**Published:** 2025-04-25

**Authors:** Theresa Hofmann, Matthias Sammer, Nadja Kohlhase, Dochka Eftimova, Felix Ehret, Antonio Santacroce, Alexander Muacevic, Christoph Fürweger

**Affiliations:** 1 Radiosurgery, European Radiosurgery Center Munich, Munich, DEU; 2 Radiation Oncology, Charité - Universitätsmedizin Berlin, Corporate Member of Freie Universität Berlin and Humboldt-Universität zu Berlin, Berlin, DEU; 3 Radiation Oncology, German Cancer Consortium (DKTK) partner site Berlin, a partnership between DKFZ and Charité – Universitätsmedizin Berlin, Berlin, DEU; 4 Neurosurgery, St. Barbara-Klinik Hamm-Heessen, Hamm, DEU; 5 Medicine, Faculty of Health, Witten/Herdecke University, Witten, DEU; 6 Stereotactic and Functional Neurosurgery, Medical Faculty, University Hospital Cologne, Cologne, DEU

**Keywords:** cyberknife, dosimetric evaluation, gyroscopic radiosurgery, robotic radiosurgery, single-fraction radiotherapy, stereotactic radiosurgery, treatment plan comparison, zap-x

## Abstract

Stereotactic radiosurgery with established systems like the Gamma Knife and CyberKnife (Accuray Inc., Madison, WI, USA) is a well-characterized treatment concept. The novel ZAP-X^®^ platform (ZAP Surgical Systems Inc., San Carlos, CA, USA) for vault-free, self-shielding gyroscopic radiosurgery (GRS) promises high plan quality due to advantageous beam properties. However, the clinically usable workspace in GRS is reduced due to potential collisions with a spacious headrest. A novel "conformal" headrest was introduced to GRS in December 2023 to remedy this, using narrower masks to minimize collision zones and maximize the usable solid angle. This study analyzes the GRS plan quality for 30 simple and complex cases, comparing GRS plans with the old and new headrests to robotic radiosurgery (RRS) as an established reference platform.

The GRS system consists of a 3 MV linear accelerator mounted on coupled gimbals for non-coplanar beam delivery, a collimator wheel for circular beam shaping, and a kV image guidance system. The RRS system is a full-body treatment platform with a 6 MV linear accelerator on a robotic arm for non-coplanar, non-isocentric beam delivery. A total of 30 clinical single-fraction plans treated with the GRS system prior to the headrest update is selected. Clinical GRS treatment plans are created by manually placing isocenters within the target volume and using an inverse optimization algorithm. GRS plans are reoptimized using the new software and headrest (further referred to as GRS*) for comparison. RRS plans are generated using circular apertures and the VOLO™ optimization technique. Treatment plans from the GRS, GRS*, and RRS platforms are compared with respect to quality metrics, number of beams, total monitor units (MU), and expected treatment time.

The updated GRS* plans show a significantly better new conformity index (nCI) and gradient index (GI) than the clinical GRS plans. The volume of the brainstem receiving 8 Gy or more is significantly reduced with the GRS* platform. The number of beams, total MU, and expected treatment time increase significantly with the new GRS* treatment planning system. Compared to GRS* plans, the nCI of RRS plans is better, but the GI is worse. The total number of beams and MU were significantly lower with the RRS platform, while the expected treatment times were equivalent.

The introduction of the new headrest design in the GRS* system has led to a notable improvement in the treatment plans of GRS. As a trade-off for the overall improvement in dosimetric quality, the number of beams and the expected treatment time increase. RRS and GRS* systems now exhibit equivalent plan quality, with a trend of the GRS* toward sharper dose gradients but lower conformity, attributed to the specialized delivery design.

## Introduction

Stereotactic radiosurgery (SRS) aims to deliver ablative doses to intracranial targets while minimizing the dose to the surrounding healthy tissue. The treatment plan characteristics of established radiosurgery systems, such as the Gamma Knife and CyberKnife (Accuray Inc., Madison, WI, USA), are well known [1-5]. Recently, the ZAP-X^®^ platform (ZAP Surgical Systems Inc., San Carlos, CA, USA) introduced vault-free, self-shielding gyroscopic radiosurgery (GRS) [6,7], featuring advantageous beam properties and potentially high plan quality [8]. Furthermore, access to a theoretical solid angle of 2 pi around the patient's head suggests the capability to achieve highly conformal plans and to flexibly shape the dose gradient [9].

However, depending on the location of the target, the clinically usable workspace in GRS is substantially reduced due to the potential collision of machine components with the spacious headrest, which is designed to hold S-type thermoplastic masks. To remedy this shortcoming, a novel, so-called “conformal” headrest was introduced to GRS in December 2023, which now uses narrower HP Pro^®^ (Orfit Industries, Wijnegem, Belgium) type masks. It is devised to minimize collision zones and maximize the usable solid angle, with the prospect of expanding the accessible patient anatomy and improving treatment plans.

Until now, GRS plan quality has only been assessed for targets of simple shape, such as brain metastases or the ventral intermediate nucleus for essential tremor [10-12]. GRS performance for complex targets is still unknown. Furthermore, the impact of the new headrest and the associated larger clinical workspace on plan characteristics has not yet been investigated.

In this study, we analyze the quality of GRS plans with the old and new headrests for 30 clinical cases. Additionally, we compare the GRS plans to robotic radiosurgery (RRS) plans from a CyberKnife as an established reference radiosurgery platform. A subset of the data was previously presented at the 2024 Radiosurgery Society Scientific Meeting.

## Technical report

Methods

Treatment Machine Characteristics

The ZAP-X^®^ GRS system comprises a 3 MV linear accelerator mounted on coupled gimbals for non-coplanar beam delivery, a collimator wheel to shape the beam circularly with eight different beam diameters from 4 mm to 25 mm in the reference distance of 45 cm, and a kV image guidance system [6,8,9,13,14]. The ZAP-X^®^ is calibrated to deliver 100 monitor units (MU) with the 25 mm field corresponding to 1 Gy at a source-axis distance of 450 mm and a depth of 7 mm in water. The ZAP-X^®^ relies on isocentric beam delivery, with each target point being moved to the machine center using the patient table. For each isocenter, the accessible solid angle depends on its location in the patient's head and associated collision zones.

**Figure 1:**
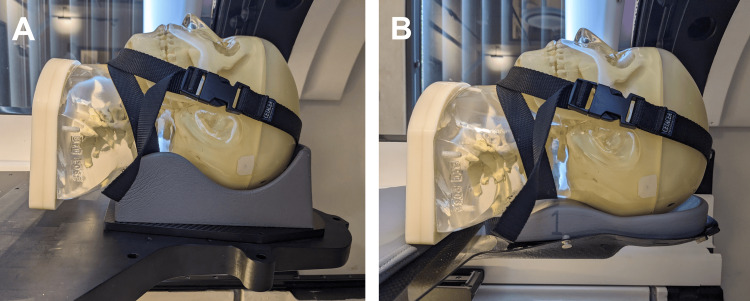
Setup of headrests with an anthropomorphic head phantom from the previous GRS (A) and updated GRS* (B) platform. GRS, gyroscopic radiosurgery

**Figure 2:**
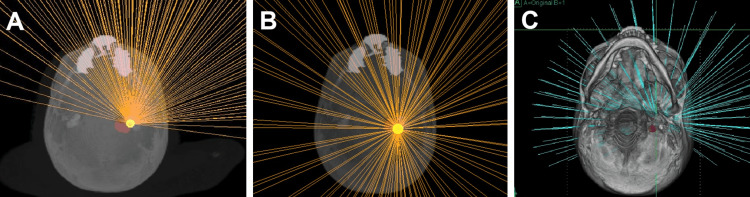
Available beams for a standard vestibular schwannoma case from the previous GRS (A), updated GRS (GRS*, B), and RRS platform (C). All images are a cross-section from the patient inferior. GRS, gyroscopic radiosurgery; RRS, robotic radiosurgery

Recently, the ZAP-X^®^ update DP1009 (treatment planning system (TPS) version 1.9.2) introduced a new patient table and conformal headrest with reduced collision zones (Figures 1, 2). We differentiate this new version using an asterisk (*) on all related variables.

The CyberKnife M6 (Accuray Inc., Madison, WI, USA) is a full-body RRS system that consists of a 6 MV linear accelerator mounted on a six degree of freedom robotic arm for non-coplanar, non-isocentric beam delivery, different collimation systems (fixed and variable circular apertures with 12 diameters ranging from 5 mm to 60 mm, and a multileaf collimation system with arbitrary aperture shapes up to 10 cm x 12 cm), and stereoscopic kV image guidance. The CyberKnife is calibrated to deliver 100 MU with the 60 mm fixed cone corresponding to 1 Gy at a source-axis distance of 800 mm and a depth of 15 mm in water.

Treatment Plan Selection

A total of 30 clinical single-fraction plans treated with the GRS system before the headrest update were selected for plan comparison. Around 19 standard vestibular schwannoma (VS) plans originated from a patient collective used in a previously reported prospective study [14]. Additionally, 337 treatments between July 2021 and September 2023 were screened for the number of isocenters used to cover the target, which represents a measure of plan complexity. A total of 11 plans with the most isocenters per target, i.e., 15 isocenters or more, were selected, including six meningiomas, three other VSs (T3b), and two arteriovenous malformations.

Treatment Plan Creation

Clinical GRS treatment plans were created by manually placing isocenters within the target volume. For each isocenter position, the TPS calculated candidate beams while avoiding potential collision with the patient. Beam weights were determined using an inverse planning algorithm, and planning goals were chosen by the planner. The overlap of isocenters caused large inhomogeneity, and the prescription isodose lines varied between 40% and 80%. The structure sets and computed tomography imaging were exported from the clinical plans created with ZAP TPS version 1.8.55 to 1.8.58 and the standard headrest. For GRS* plans with the new update DP1009, the original isocenter positions were retained, and the plans were reoptimized in the new software, assuming the use of the new conformal headrest. The software calculated the expected treatment time, which included an initial setup time of 10 minutes. We subtracted this setup time so that the expected treatment time only includes gantry and table motion, high voltage on time, beam on time, kV imaging between isocenters, and computational time. RRS plans were created in the Precision^®^ TPS (version 3.4.0.0), using circular apertures and the VOLO™ optimization technique [15]. With the RRS, the TPS first calculated candidate beams from predefined nodes directed to a region of the target volume, then inversely optimized the beam weights according to goals selected by the planner. The RRS TPS calculated an expected treatment time, which starts after the initial setup. In all clinical and comparison plans, both eyes were blocked for beam passage, and the dose to organs at risk was kept as low as possible while still allowing for high target coverage. We used the same constraints for the upper limits for organs at risk, i.e., D_max_, optic system < 6 Gy, V8Gy, brainstem < 1 cm^3^, and D_mean_, cochlea < 4 Gy, which were internal limits supported by literature [16,17]. Heterogeneity was not penalized. The GRS* and RRS treatment plans for each case were renormalized to match the coverage of the clinical GRS plan to within ±0.2%. The same path densities were used for each isocenter in GRS and GRS* plans.

Treatment Plan Comparison

Treatment plans were compared with respect to dosimetric quality measures such as mean target dose, the new conformity index (nCI), and the gradient index (GI), as well as the number of beams, total number of MU, and expected treatment time calculated from the TPS [18,19]. Additionally, cochlea dose (mean and maximum) and brainstem volume receiving 8 Gy or more were compared.

The nCI describes the conformality of the prescription dose and is defined as

$\text{nCI} = \frac{\text{TV} \cdot \text{PIV}}{\text{TV}_\text{PIV}^2}$,

where TV is the tumor volume, PIV is the total tissue volume receiving the prescription dose or more, and TV_PIV_ is the tumor volume receiving the prescription dose or more.

The GI describes the gradient of the dose fall off and is defined as

$\text{GI} = \frac{\text{hPIV}}{\text{PIV}}$,

where hPIV is the total tissue volume receiving half the prescription dose or more and PIV, as defined above.

A Wilcoxon signed rank test evaluated differences between TPS versions and machines, with a significance level of α=0.05 and no correction for multiple testing. Statistical analyses were performed using Python SciPy (version 1.6.2, https://scipy.org) [20].

Plans from the updated GRS* platform were compared to clinical GRS plans. These plans were then compared to RRS plans, which served as the reference.

Results

Clinical Treatment Plan Characteristics

**Figure 3:**
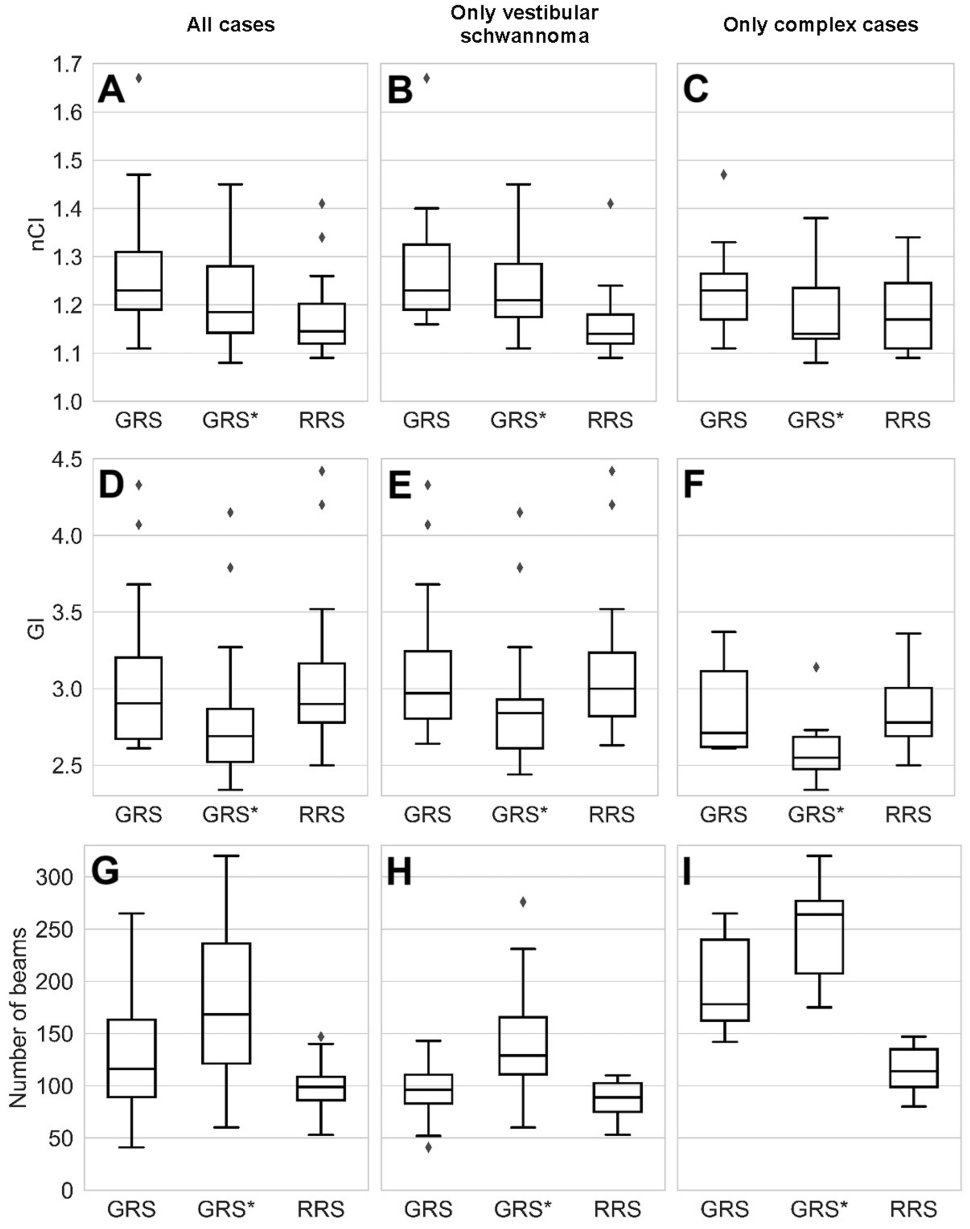
Histograms of the nCI (first row: A-C), GI (second row: D-F), and number of beams (third row: G-I) for all plans in the analysis (first column: A, D, G), vestibular schwannoma cases (second column: B, E, H) and complex cases (third column: C, F, I), calculated with the GRS TPS, the updated GRS TPS (GRS*), and the RRS TPS. GI, gradient index; GRS, gyroscopic radiosurgery; nCI, new conformity index; RRS, robotic radiosurgery; TPS, treatment planning system

**Figure 4:**
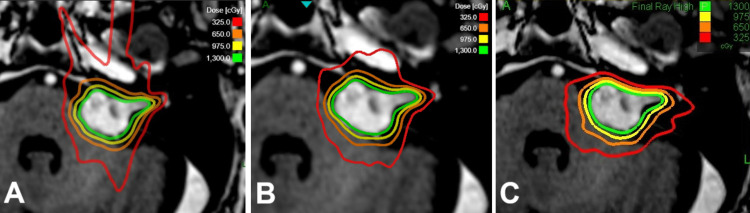
Example of three plans for a complex vestibular schwannoma case. (A) Clinical treatment plan with GRS prior to the update. (B) Plan calculated with the updated GRS system (GRS*). (C) Plan calculated with RRS. GRS, gyroscopic radiosurgery; RRS, robotic radiosurgery

Out of the 30 patients, 17 were male and 13 were female. The median age at the time of treatment was 62 years (range 28 to 81 years). The target volumes ranged from 0.09 cm^3^ to 8.69 cm^3^, with a median of 1.57 cm^3^. The prescription dose ranged from 13 Gy to 18 Gy (13 Gy to 13.5 Gy for VSs, 16 Gy to 17 Gy for arteriovenous malformations, and 14 Gy to 18 Gy for meningiomas). In the clinical GRS plans, the prescription isodose line ranged from 41% to 62%. The target coverage ranged from 93.2% to 99.5%, with a mean target dose of 16.0 Gy to 22.8 Gy. The median nCI was 1.23 (range 1.11 to 1.67), and the median GI was 2.91 (range 2.61 to 4.33), see Figures 3A, 3D, respectively. The volume of the brainstem receiving 8 Gy or more varied between 0.0 cm^3^ and 1.4 cm^3^, with a median of 0.02 cm^3^. In almost half (14) of the 30 cases, this volume was exactly 0.0 cm^3^. In two out of the 30 cases, the dose constraint of 1.0 cm^3^ was exceeded, with 1.1 cm^3^ and 1.4 cm^3^. In these two cases, the target volume was directly adjacent to the brainstem. For VS cases, two patients were deaf before the treatment, and, therefore, the dose to the cochlea was not evaluated. For the remaining patients with hearing function, the mean cochlea dose was between 0.7 Gy and 5.3 Gy, with a median of 3.4 Gy. The maximum cochlea dose was between 1.7 Gy and 12.3 Gy, with a median of 7.1 Gy. In terms of delivery parameters, the number of isocenters ranged from 2 to 23, while the number of beams ranged from 41 to 265 (Figure 3G). The number of MU was between 6,475 and 25,446. The expected treatment time was between 6 and 65 minutes, excluding setup time. The treatment plan and patient characteristics are presented in Table 1. An example of a treatment plan for a complex case is shown in Figure 4A.

**Table 1:** Treatment plan characteristics from the three different TPS: GRS (pre-update), GRS* (post-update), and RRS platform. Values are given as median (min–max). Dose metrics given for the cochlea correspond to the vestibular schwannoma cases with hearing function only. GI, gradient index; GRS, gyroscopic radiosurgery; nCI, new conformity index; p, p-value of Wilcoxon rank test; RRS, robotic radiosurgery; TPS, treatment planning system

	Metric	GRS	p_GRS-GRS*_	GRS*	p_GRS*-RRS_	RRS
Prescription	Dose (Gy)	13 (13–18)		13 (13–18)		13 (13–18)
	Isodose (%)	52 (41–62)		51.6 (43.2–67.4)		51.6 (46.1–67.6)
Target	Coverage (%)	97.6 (93.3–99.5)	0.20	97.6 (93.1–99.4)	0.83	97.6 (93.2–99.4)
	Mean dose (Gy)	17.9 (16.0–22.8)	0.08	18.2 (15.7–23.9)	0.10	17.9 (15.4–22.1)
	nCI	1.23 (1.11–1.67)	9e-3	1.19 (1.08–1.45)	2e-3	1.15 (1.09–1.41)
Global	GI	2.91 (2.61–4.33)	4e-6	2.69 (2.34–4.15)	2e-6	2.90 (2.50–4.42)
Cochlea	Max dose (Gy)	7.1 (1.7–12.3)	8e-3	5.4 (1.0–12.1)	0.09	6.1 (2.1–12.4)
	Mean dose (Gy)	3.4 (0.7–5.3)	0.37	2.9 (0.5–4.6)	0.23	2.6 (1.4–3.9)
Brainstem	V8Gy (cm³)	0.02 (0.0–1.4)	4e-3	0.01 (0.0–1.4)	0.11	0.01 (0.0–1.0)
Delivery	Beams	116 (41–265)	2e-6	169 (60–320)	3e-6	99 (53–147)
	Monitor units (1000)	11.8 (6.5–25.4)	0.04	12.7 (6.3–24.8)	2e-5	9.8 (5.2–17.1)
	Estimated treatment time (min)	25 (6–65)	3e-5	33 (11–70)	0.08	31 (19–47)

Comparison of Treatment Plans With the Updated GRS*

Characteristics of the updated GRS* plans are summarized in Table 1. While there was no significant difference in mean target dose (p=0.08), the updated GRS* plans showed a significantly smaller nCI compared to the clinical GRS plans, with a median of 1.19 (range 1.08 to 1.45, p=0.009, Figure 3A). Also, the GI was significantly smaller than the one of the clinical plans (median 2.69, range 2.34 to 4.15, p<0.001, Figure 3D). The volume of the brainstem receiving 8 Gy or more was significantly reduced in the GRS* platform (p=0.004). The two cases exceeding 1 cm^3^ were slightly improved (from 1.4 cm^3^ to 1.37 cm^3^ and from 1.14 cm^3^ to 1.03 cm^3^) but still exceeded the 1 cm^3^ constraint. For VS cases, there was a significant difference in the maximum dose to the cochlea (p=0.008). Conversely, no statistically significant difference could be ascertained for the mean dose to the cochlea (p=0.37). In return to the dosimetric quality improvement, the number of beams (p<0.001), the total number of MU (p=0.04), and the expected treatment time (p<0.001) increased significantly with the new GRS* TPS. With the new GRS* platform, the plans had a median number of beams of 169 (range 60 to 320, Figure 3G), total MU of median 12,719 (range 6,337 to 24,776), and a median expected treatment time of 33 minutes (range 11 minutes to 70 minutes). An example of a comparison treatment plan with the GRS* platform is shown in Figure 4B. 

Comparison of Treatment Plans With the Updated GRS* to RRS Plans

Between the plans from the RRS and GRS* platforms, there was no significant difference in mean target dose (p=0.10). The nCI of RRS plans was lower, with a median value of 1.15 (range 1.09 to 1.41, p=0.003, Figure 3A). Conversely, the median GI of 2.90 of the RRS platform (range 2.50 to 4.42) was significantly larger than the GI of 2.69 of the new GRS* platform (p<0.001, Figure 3D). There was no significant difference (p=0.11) between the volumes of the brainstem receiving 8 Gy or more. However, the two cases exceeding the 1 cm^3^ volume constraint were below or right at this limit in the RRS platform (1.0 cm^3^ and 0.87 cm^3^, respectively). Also, the maximum and mean dose to the cochlea did not show a significant difference between the GRS* and RRS plans (p=0.09 and p=0.23, respectively). The total number of beams per treatment was significantly lower with the RRS platform (median 99, range 53 to 147, p<0.001, Figure 3G), as well as the total number of MU (median 9,822 MU, range 5,204 MU to 17,058 MU, p<0.001). Still, the expected treatment time showed no significant difference between the RRS and GRS* platforms, with a median of 31 minutes (range 19 minutes to 47 minutes, p=0.08) for the RRS. Characteristics of the RRS plans and corresponding p-values are displayed in Table 1. An example of a comparison treatment plan created with the RRS platform is shown in Figure 4C.

Separate Analysis of VS (Standard) Cases and Complex Cases

When the two groups of standard (i.e., VS) and complex cases were evaluated separately, most of the trends remained the same (see Figure 3 for a comparison of histograms of the nCI, GI, and number of beams). We will, therefore, only point out differences here. For the group of complex cases, there was no significant difference in MU between the GRS* and GRS platforms. When comparing GRS* to RRS plans, there was no significant difference in the nCI for these complex cases (Figure 3C). However, the expected treatment time for complex cases was significantly larger for plans from the GRS* platform in comparison to the RRS platform. For standard VS cases only, there was no significant improvement in the nCI on the GRS* platform in comparison to the clinical GRS plans (p=0.21, Figure 3B). The mean target dose was significantly larger and the mean cochlea dose was significantly lower in VS cases on the GRS* platform in comparison to the plans from the RRS platform.

## Discussion

Vault-free GRS is a novel technique specifically designed for treating intracranial targets while minimizing the impact on surrounding healthy tissues. Since its introduction and first installation in Europe in 2021, substantial clinical experience has been gained in treating patients using the system. One limiting factor of the GRS system has been the headrest of the patient table, which is designed with generously large dimensions. While the headrest's substantial size enhances patient stability and accessibility of the patient mask, it restricts the available irradiation directions due to collision safety zones. Consequently, a new headrest design was introduced with the ZAP-X^®^ TPS update (DP 1009), allowing for more beam directions similar to those of an RRS platform. In this study, clinical GRS treatment plans from 30 patients are compared to reoptimized plans using the updated GRS* version and the RRS, which is a well-established treatment modality in radiosurgery.

Comparison of treatment plans between the GRS and the updated GRS*

The latest headrest update (DP 1009) from the GRS* platform represents a significant improvement in the quality of treatment plans. Although the mean target dose in GRS* plans does not show a significant difference, the updated GRS* plans demonstrate a notable improvement in the GI compared to the original GRS plans. While there is no significant difference in the nCI for standard cases, complex cases show a significant improvement in the nCI as well. The updated GRS* significantly reduces the brainstem volume receiving 8 Gy or more by 0.1 cm³ for the median. Two cases exceed its volume constraint of 1 cm³ and are slightly improved by the updated GRS* (from 1.4 cm³ to 1.37 cm³ and from 1.14 cm³ to 1.03 cm³), while the constraint is still not met. For VS cases, there is no significant difference in the mean dose to the cochlea between the GRS and GRS* versions, but a significant difference can be observed for the maximum dose to the cochlea. As a trade-off for general dosimetric quality improvement, the number of beams, total number of MU, and treatment time increase significantly with the new GRS* TPS by 53 beams, 900 MU, and 8 minutes for the median, respectively. In both the GRS and GRS* TPS, the availability of more beams leads to a higher number of beams in the final plan. Therefore, the introduction of the new headrest, which offers more beam angles per isocenter, likely explains the increased number of beams in the final GRS* plans. This, in turn, results in longer treatment times. The difference in MU originates in the VS cases only, while complex cases do not show this significant trend. The latter increases are deemed clinically acceptable, given the benefits associated with the enhanced dosimetric parameters.

Comparison between GRS* and RRS plans

The RRS is a well-established treatment modality in radiosurgery. We evaluate the quality of GRS* treatments by comparison to RRS plans as a benchmark. While the nCI of GRS* plans for standard cases is significantly higher, the GI of all GRS* plans is significantly lower than the one of RRS plans. The better conformity of the RRS plans might be a result of the non-isocentric delivery, which is currently not available on the GRS* system [4,10,21]. The sharper dose gradient of the GRS* system is attributed to its specialized delivery design, which includes lower beam energies from the 3 MV linear accelerator, smaller collimators (down to 4 mm compared to 5 mm in the RRS platform), and optimized geometric relationships between the focal spot, collimator, and isocenter within the GRS system. These factors collectively result in sharper penumbras, enhancing the system's potential gradient [22,23]. Often, a dedicated SRS multileaf collimator is used for complex shapes on the RRS platform, offering a potential benefit in these cases for conformity, GI, and treatment time. Due to the different properties of a multileaf collimator and comparability to other plans, this aspect has not been evaluated in this study and is beyond the scope of this paper.

Concerning organs at risk examined in the study, there is no significant difference between the volumes of the brainstem receiving 8 Gy or more in the RRS and GRS* platforms. However, in the two cases where the 1 cm^3^ volume constraint is exceeded, the RRS platform managed to keep the volumes below or at this limit. For standard VS cases only, the mean cochlea dose is significantly lower on the GRS* platform in comparison to the RRS platform. This does not hold for the complex VS cases, although there are only three of them. This phenomenon could be explained by a sharper dose fall-off available to the cochlea using the 4 mm collimator and more available beam angles in comparison to the scenario prior to the update.

The RRS platform has a significantly lower total number of beams per treatment and MU compared to the GRS* platform. The difference in the number of MU originates in the different beam energies (3 MV on the GRS* versus 6 MV on the RRS) and depth dose distributions between the two systems. While the total number of MU is an easy-to-measure parameter, it does not necessarily translate into treatment time due to the different energies and dose rates of the systems. Since the dose rate of the GRS* is almost twice as high as the one from the RRS platform (1500 MU/min versus 800 MU/min, respectively), the beam on time on the GRS* is still lower than on the RRS platform, which partially compensates for the increased number of MU and beams in the calculation of expected treatment time. Therefore, the expected treatment time is not significantly different between the two platforms for standard cases. For complex cases, the expected treatment time was found to be higher on the GRS* platform. This can be at least partially explained by an inherent selection bias, which was introduced by defining complex cases as the ones with most GRS isocenters used per target. Intrinsically, additional isocenters introduce extra time for shifting and aligning the patient, leading to a higher expected treatment time. Due to the increased number of beams per isocenter and a larger solid angle in the GRS*, this expected treatment time might be reduced with fewer isocenters and a potentially easier way to reduce the number of beams in the TPS in the future.

Limitations and future perspectives

While this study compares plans for GRS and RRS, some aspects have been neglected so far. As mentioned before, a multileaf collimator is available for the RRS platform, potentially increasing conformity, GI, MU, and treatment time, especially for cases with targets larger than 2 cm [24,25]. Also, during delivery, the GRS platform, until DP 1010, is able to correct for translational offsets only, while the RRS platform corrects for six degrees of freedom. This leads to a slightly larger uncertainty during treatment delivery but has not been studied herein. The next update of the GRS platform (DP 1011) introduces a six-degree freedom correction during delivery, potentially making up for this disadvantage.

Further, it is important to acknowledge the potential bias introduced by user dependence, which is inherent to a single-center study. A multicentric comparison might provide a more comprehensive assessment of the potential of GRS but also introduces challenges due to different clinical practices and priorities [26].

We reported equivalent treatment times for RRS and GRS platforms, which did not include patient setup. In our experience, this setup takes longer on the GRS platform for two reasons. First, the single X-ray tube requires gantry movement to acquire images from different angles, which are necessary for proper patient setup. Second, the limitation of the couch to correct the patient only in three dimensions results in occasional need to manually reposition the patient’s head. This prolongs the total treatment process on the GRS but has been neglected in this study.

Another limitation of the plans on the GRS platform is the manual isocenter placement. Depending on the experience of the treatment planner, the location of isocenters can be suboptimal. This has large effects on the plan quality and could potentially be improved and standardized in future versions of the system [27].

## Conclusions

The introduction of the new headrest design in the GRS* system has led to a significant improvement in treatment plans in terms of conformity and dose gradient. As a trade-off for the overall improvement in dosimetric quality, the number of beams and the expected treatment time increased significantly. Compared to the RRS system, the GRS* system demonstrated equivalent plan quality, with a statistically significant trend toward sharper dose gradients but lower conformity, attributed to the specialized delivery design.
